# Evolution of aging through reduced demographic stochasticity – an extension of the pleiotropy theory to finite populations

**DOI:** 10.1002/ece3.889

**Published:** 2013-12-21

**Authors:** Stefano Giaimo

**Affiliations:** Max Planck Research Group Modeling the Evolution of Aging, Max Planck Institute for Demographic ResearchKonrad Zuse Strasse 1, 18057, Rostock, Germany

**Keywords:** Aging, demographic variance, evolution, pleiotropy, senescence, stochasticity

## Abstract

In finite populations, there is selection against demographic stochasticity. In this study, it is shown that an increase in the rate of aging, here defined as an increase in early-life survival at the expense of later survival, may reduce this form of stochasticity. In particular, a trade-off between juvenile and adult survival is highly efficient in reducing demographic stochasticity. Therefore, aging may evolve as a response to selective pressure for reduced demographic stochasticity.

## Introduction

Random variation between independent, individual events introduces demographic stochasticity in the population, which does not grow deterministically but fluctuates in size. When the population is age-structured, the proportions of individuals that enter the first age class and flow from one age class to the next are not constant. Demographic stochasticity is inevitable and independent from the environment, as individual survival and reproduction are intrinsically probabilistic events. In an infinite population, one can neglect demographic stochasticity, as individual variations average out. However, in a finite population, they are less likely to do so. Therefore, the effect of demographic stochasticity cannot be ignored.

Demographic stochasticity has been shown to play a crucial role in many evolutionary and ecological dynamics of finite populations (Lande et al. 2003; Engen et al. 2005a,b2005b; Shpak 2007; Vindenes et al. 2009). In particular, it may be an important factor in deciding the ultimate fate of an allele newly arisen by mutation. Gillespie (1974) considers a haploid model with a constant environment and no age structure. He derives for this model an approximation to the fixation probability of a mutation with the same expected number of offspring as the wild type but different variance. In Gillespie's model, the mutant and the wild-type subpopulations are subject to different amounts of demographic stochasticity, as this is proportional to the individual variance in offspring number. Gillespie shows that, independently of the exact population size, if the mutation has smaller variance than the wild type, then it has a selective advantage; if the mutation has greater variance than the wild type, then it has a selective disadvantage. This model shows the existence of natural selection against demographic stochasticity. Intuitively, a genotype that always leaves one offspring is fitter than a competing genotype that leaves either zero or two offspring with equal probability. The two genotypes have the same expected number of offspring. However, the first genotype has no variance in offspring number and no risk of going extinct, as it is always sure to deliver one offspring. The second genotype has some variance in offspring number, which puts it at risk of extinction – that is, there is always a nonzero probability 1/2^*N*^ that all *N* individuals that compose this genotype subpopulation simultaneously deliver zero offspring. More formally, the selective advantage *s* for a mutation that only differs from the wild type in having reduced variance in offspring number *σ*^2^ can be expressed as


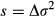
(1)

Through analytic and simulation arguments, Shpak (2007) generalizes this result by Gillespie to finite populations with age structure assuming no environmental variability. He interprets the variance term in the above equation as the demographic variance of competing haploid genotypes that have equal growth rate. He shows that the genotype with smaller demographic variance than its competitor has a selective advantage. The demographic variance (more on this below) captures the amount of demographic stochasticity in an age-structured model when the population growth rate is not too different from unity (Lande et al. 2003). The demographic variance is the variance in the population growth rate (measured on the log scale) due to the variance in, and covariance among, age-specific individual survival and fertility in a constant environment (Engen et al. 2005b).

In the present study, I propose that equation (1) can be applied to the evolutionary theory of aging. I assume a constant environment and asexual haploid populations. I consider mutations that increase early-life survival at the expense of later survival but hold the growth rate constant. I assume that the population growth rate is not too different from unity. I put forth a heuristic argument to the effect that mutations of this sort, which are supposed to drive the evolution of aging (Williams 1957), may reduce the demographic variance and therefore should be selected for. I validate this heuristic argument with computer simulations. Therefore, I suggest aging may be a response to selective pressure for reduced demographic stochasticity.

### Demographic variance

Consider a haploid population with age structure in a constant environment. The characteristics of this population can be modeled by a prebreeding census Leslie matrix:


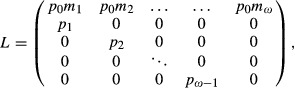
(2)

(Caswell 2001). In this matrix, *p*_*x*_ and *m*_*x*_ are the age-specific individual survival and fecundity, respectively. The leading eigenvalue *λ* of *L* is the growth rate of the population and is the implicit solution of the characteristic equation


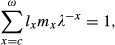
(3)

where *l*_0_ = 1 and *l*_*k*_ = *p*_0_*p*_1_*p*_2_…*p*_*k*−1_; and *c* is the age class of first reproduction (Charlesworth 1980). In this article, it is assumed that *λ *≈ 1. In the absence of covariance among the parameters of *L*, the demographic variance of this population is


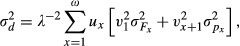
(4)

where *v*_*ω*+1_ = 0 (Engen et al. 2005b). In this equation, *u*_k_ and *v*_k_ are the elements of the appropriately scaled right and left eigenvectors of *L* (corresponding to *λ*), respectively. The right eigenvector *u* of *L* is scaled so that


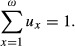
(5)

Thus, *u*_k_ is the stable proportion of individuals in age class *k* and is equal to


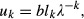
(6)

where 
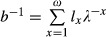
. The left eigenvector *v* of *L* is scaled so that


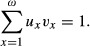
(7)

With this scaling, *v*_*k*_ is the average reproductive value of individuals in age class *k* and is equal to the discrete version of Fisher's individual reproductive value (Fisher 1930):


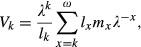
(8)

divided by *Tb* with 
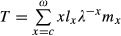
, which is the usual measure of generation time. The relation *v*_*k*_ = *V*_*k*_/*Tb* follows immediately from the fact that 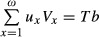, as shown in Felsenstein (1971). In the demographic variance, 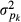 and 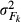 are the variances in *p*_*k*_ and *p*_0_*m*_*k*_, respectively. Therefore, 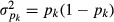, while the form of 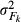 depends on how *m*_*k*_ is modeled. In this study, it is assumed that *m*_*k*_ is the expected value of the random variable *w*_*k*_ *b*_*k*_, where *w*_*k*_ is a Bernoulli random variable and *b*_*k*_ a strictly positive constant. Here, I assume that *m*_*k*_ is independent from p_0_ and that *b*_*k*_ = 1 in all reproductive age classes. Therefore, 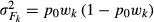. Biologically, these assumptions about fecundity correspond to long-lived vertebrates with a relatively long generation time that have no more than one offspring per season (Harvey et al. 1989; Sæther and Bakke 2000; Engen et al. 2005b). Populations of this sort are the target of the present article. However, for simplicity, I neglect sex and diploidy. Based on these assumptions, the demographic variance is rewritten as


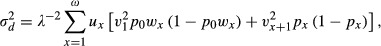
(9)

which corresponds to the right-hand side of equation (13) in Engen et al. (2005b).

### Pleiotropy for survival

If we split the main sum of equation (9), we can express the demographic variance as 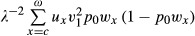 plus 
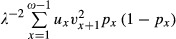
. If both *c* and *p*_0_ are relatively high, then we can hypothesize that the former quantity will be relatively small, and that the demographic variance will be determined mainly by the latter quantity. By noting that *λ*^−1^*u*_*k*_*p*_*k*_ = *u*_*k*+1_, this quantity can also be expressed as


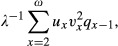
(10)

where *q*_*k*_ = 1−*p*_*k*_. We now consider pleiotropy for survival. Suppose that a mutation increases *p*_*i*_ and at the same time decreases *p*_*j*_. Assume that 0 < *i* < *j* and that lambda holds constant. The assumption 0 < *i* is made to avoid changes in fertility variance 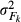. Equation (6) suggests that the effect of the mutation on *u*_*k*_ is an increase in and between the age classes (*i* + 1) and *j*, because survivorship *l*_*k*_ is increased in these age classes in the mutant. As equation (5) must hold, *u*_*k*_ is decreased in all other age classes as a result of a decrease in *b* and, if *j* is not a prereproductive age class, of a decrease in survivorship in age classes greater than *j*. We can expect the opposite pattern of effects on *v*_*k*_. This is suggested by the presence of the factor 1/*l*_*k*_ in equation (8). Reproductive value is decreased in and between the age classes (*i* + 1) and *j*. But *v*_*k*_ is increased in all other age classes, as equation (7) must hold or, equivalently, we should remember that *v*_*k*_ = *V*_*k*_/*Tb*, and *T* and *b* will be decreased by the mutation (note, however, that *T* holds constant if *j* is a prereproductive age class). Less formally, the mutation increases the proportions of individuals within certain age classes at the expenses of the proportions in other age classes. The increase occurs in and between the age classes (*i* + 1) and *j*. In order to keep the population growth constant, a compensation process takes place: the reproductive value of age classes with increased representation must decrease, while the reproductive value of age classes with decreased representation must increase. Let us put aside the opposite age-specific mutational effects on *q*_*i*_ and *q*_*j*_. It is likely that the overall effect of the mutation on the demographic variance is determined more by its effect on the reproductive value than by its effect on the stable age distribution. This is because *v*_*k*_ but not *u*_*k*_ appears squared in the demographic variance; see equations (9) and (10). However, we should keep in mind the trajectory of *v*_*k*_ for typical life histories. The reproductive value increases from birth up to a peak around the age of first reproduction and then progressively decreases (Caswell 2001). We expect the mutant reproductive value to increase in age classes before (*i* + 1) and after *j*. If both *i* and *j* are either prereproductive or reproductive age classes, then the mutation will provoke an increase in reproductive value at the age classes in which reproductive value reaches its maximum. In contrast, if *i* and *j* denote a prereproductive and a reproductive age class, respectively, then the mutation is expected to have the greatest impact on the overall diminution of reproductive value, particularly, when *i* and *j* are relatively distant. Therefore, it is under the condition 0 < *i* < *c* < *j* (or, more generally, when a high concentration of reproductive value is contained between ages *i* and *j*) that we should expect a reduction in the mutant demographic variance.

### Simulations

The above heuristic argument is validated by simulations. 100,000 life histories are generated with *ω* = 16. *p*_0_ is set to 0.8. For all *p*_*k*_ such that 0 < *k* < *c*, the value 0.95 is set. *m*_*c*_ is set 0.9. For *c* ≤ *k* ≤ (*ω*−1), the value of *p*_*k*_ and the value of *m*_*k*_ are separately drawn at random from the set {0.9; 0.85; 0.8; 0.75; 0.7; 0.65; 0.6; 0.55; 0.5; 0.45; 0.4; 0.35; 0.3}. The conditions are imposed that *p*_*k*_ ≥ *p*_*k* + 1_ and *m*_*k*_ ≥ *m*_*k* + 1_. This implies the absence of aging or the presence of aging to some degree but no negative aging. Life histories with *λ *< 1 and life histories that represent copies of others that are already generated are removed from the pool. Each of the remaining life histories is mutated in the following way. A positive quantity (*π*) is added to a randomly selected survival probability *p*_*i*_ with 0 < *i* < (*ω*−1). A randomly selected survival probability *p*_*j*_ with *j* > *i* is subtracted a sufficient quantity *ε* to restore the value of *λ* prior to the increase in *p*_*i*_. If (*p*_*j*_−*ε*) < 0, the life history is removed from the pool. For each life history, the value of the demographic variance is compared before and after the mutation. At the end, the recorded data are how many life histories were not removed from the pool, whether or not the demographic variance of these life histories is smaller after the mutation, whether or not *i* < *c* < *j*, and whether or not *j* > *c*. The effect of different values of *c* and *π* is considered. Results are reported in Table 1. Overall, a reduction in the demographic variance is very likely after a mutation with pleiotropic effects on survival. When *i *< *c* < *j*, a reduction in the demographic variance is practically certain. Failure to reduce the demographic variance is almost fully explained by cases in which *j* < *c*. This also explains the decreasing percentage of reduction in the demographic variance found with increasing age of first reproduction: the random selection of age class *j* is more likely to pick up a prereproductive age class because the juvenile stage is longer. The magnitude of the mutation does not appear to play a role. In general, it is very likely that a pleiotropic mutation reduces the demographic variance when its negative effect is exerted at reproductive age classes (*j* > *c*). The likelihood of the reduction is extremely high when the trade-off is between juvenile survival and adult survival. Another set of simulations is performed to test the robustness of this result by applying the following modifications to the above procedures: 500,000 life histories are generated; *c* is set to 2; *p*_0_ is set to 0.65; for 0 < *k* ≤ (*ω*−1), the value of *p*_*k*_ is drawn at random from the set {0.9; 0.85; 0.8; 0.75; 0.7; 0.65; 0.6; 0.55; 0.5; 0.45; 0.4; 0.35; 0.3} with the condition that *p*_*k*_ ≥ *p*_*k* + 1_ but note that the value of p_1_ is drawn independently from *p*_0_; and *π* is set to 0.001. With these settings, newborn and juvenile survival is not assumed to be high, and the juvenile stage is short. The effect of a change in the maximum age class is explored. When *ω* = 4, all mutated life histories (2751) get a reduced demographic variance, while when *ω* = 8, only a fraction (∼65.222%) of all mutated life histories (11,398) do so. When attention is restricted to the case in which *i* < *c* < *j* (here *i *= 1 necessarily), all mutated life histories obtain a reduction in the demographic variance independently of *ω* (673 for *ω* = 4; 1409 for *ω* = 8). On the one hand, these results confirm the extremely high likelihood that a trade-off between juvenile survival and adult survival decreases the demographic stochasticity. On the other hand, they show a marked difference between *ω* = 4 and *ω* = 8. This difference is almost certainly due to cases in which both *i* and *j* are reproductive age classes, as only a small fraction of life histories have *i* < *c* < *j*. The reason behind the low proportion of cases in which *i* < *c* < *j* appears to be that, when the juvenile stage is short and the adult stage is long (*c* = 2 and *ω* = 8), it is much more likely that the random choice of *i* picks on a relatively late adult age. But reproductive value is very low at late reproductive ages, especially under the assumptions made here. The reduction in reproductive value – which occurs between *i* and *j* as a result of the mutation – is very likely to be outweighed by its increase at earlier age classes, in which reproductive value is high. Overall, the obtained results suggest that, unless *j* < *c* (mutant survival disadvantage at prereproductive ages) or *i *≫ *c* (mutant survival advantage at late ages), a mutation that increases early-life survival at the expense of later survival is very likely to reduce the demographic variance. This effect is mediated by a reduction in reproductive value in the age classes in which this is most concentrated, at least in typical life histories. In virtue of equation (1), the mutation should confer increased fitness.

**Table 1:** Effect of pleiotropy for survival on the demographic variance 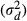 at different ages of first reproduction (*c*) and different magnitudes (*π*) of the beneficial effect of the mutation (at age *i*).

*c*	*π*	No. of life histories	No. of life histories with reduced 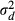	No. of life histories with *i* < *c* < *j*	% reduced 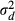 with *i* < *c* < *j*	No. of life histories with *j* < *c*	% reduced 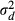 with *j* < *c*
4	1.00 × 10^−5^	36,708	36,082	5518	100	624	0
	1.00 × 10^−4^	33,045	32,423	4316	100	621	0
	0.001	27,420	26,780	3036	100	636	0
	0.01	18,626	18,056	1880	100	567	0
6	1.00 × 10^−5^	24,213	22,863	6191	100	1340	0
	1.00 × 10^−4^	22,439	21,076	5101	100	1357	0
	0.001	18,766	17,415	3687	100	1335	0
	0.01	13,712	12,310	2329	100	1392	0
8	1.00 × 10^−5^	13,196	11,601	4273	∼100	1573	0
	1.00 × 10^−4^	12,804	11,150	4071	100	1635	0
	0.001	11,437	9793	3089	100	1623	0
	0.01	8679	7086	1934	100	1566	0
10	1.00 × 10^−5^	5359	4147	1870	100	1184	0
	1.00 × 10^−4^	5415	4135	1848	100	1235	0
	0.001	5294	3961	1795	100	1295	0
	0.01	4492	3240	1273	100	1221	0

## Discussion

It is well known that stochastic variations in population growth may play a crucial role in life-history evolution (Caswell 2001; Lande et al. 2003; Tuljapurkar et al. 2009). Aging, here defined as the progressive decrease in survival with age (Comfort 1979; Finch 1994), is an important life-history trait. Orzack and Tuljapurkar (1989) studied the evolution of aging under environmental stochasticity but without demographic stochasticity. They compared the fitness of life histories with different degrees of iteroparity (reproducing many times through life) when changes in the environment cause the vital rates to fluctuate randomly through time. They showed that when environmental variability is high, selection favors a high level of iteroparity (prolonged life span and extended reproduction); when environmental variability is low, selection favors a low level of iteroparity (reduced life span and a shorter but more fecund reproductive period) (Orzack and Tuljapurkar 1989). In their analysis, fitness is approximated by 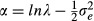 (Tuljapurkar 1982). Here, 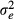 is the environmental variance of the population, which is the sum of variances in expected individual fitness in a given age induced by changes in the environment.

In the present study, the assumptions of Orzack and Tuljapurkar are reversed: The environment is constant 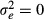, and the population is subject to demographic stochasticity exclusively. Following Shpak (2007), the fitness of different life histories with equal growth rate *λ* (≈1) is assumed to be inversely proportional to the value of the demographic variance 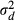. This is the sum of expected variances in individual age-specific fitness in the population. (Engen et al. 1998, 2005b, 2009; Lande et al. 2003) discuss in detail the conceptual and mathematical differences between 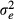 and 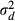 The present work shows that mutations with a positive effect on early-life survival at the expense of later survival are very likely to reduce the demographic variance and therefore to be fitter than the wild type, particularly, when the trade-off is between prereproductive and reproductive ages. The finding that, in a constant environment, selection for reduced demographic stochasticity may favor aging shows a potential continuity with the result of Orzack and Tuljapurkar (1989) that reduced life span and compressed reproduction may be selectively advantageous under low environmental variability. Therefore, it seems plausible that a higher rate of aging should be expected to evolve as the environment gets less and less variable and, eventually, becomes constant. Instead, when the environment is highly variable, we have two antagonistic forces: selection for a higher rate of aging through reduced demographic variance (the result of the present study) and selection for a lower rate of aging through increased alpha (Orzack and Tuljapurkar 1989). The existence of a contrast between selection for a more iteroparous life history in a highly variable environment and selection for a less iteroparous life history in a stable or scarcely variable environment can probably be explained as follows. When the environment strongly changes with time, it may make sense for an organism to go through several successive reproduction events, because at least some of them will have the chance of occurring under favorable environmental conditions. Conversely, when the environment is stable, it is not a sensible choice to “wait” for the right time to reproduce, because every moment is equally favorable. Therefore, it is preferable to concentrate one's reproductive chances into a short time after maturation (i.e., as soon as one can reproduce) or around the peak of reproductive value, because in this situation, the worst enemy of successful reproduction is the risk of death from general causes (i.e., unrelated to fluctuations in the environment) before the full reproductive potential is delivered.

However, the impact of demographic stochasticity on evolutionary dynamics is inversely proportional to population size (Lande et al. 2003). Indeed the expected growth rate of a population subject to both demographic and environmental stochasticity is 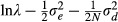, where *N* is the population size (Engen et al. 2005b). Lande et al. (2003) suggest that environmental stochasticity is much more important to evolutionary dynamics than demographic stochasticity when 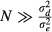. They estimate that this inequality typically holds for natural populations that are subject to both types of stochasticity when their size is greater than 100. Therefore, mainly small populations that are exposed to no, or very little, environmental variability are expected to show a higher rate of aging. As for populations exposed to high environmental variability, it should be kept in mind that simulations studies (Koons et al. 2009) have suggested that selection may not necessarily favor a reduction in environmental stochasticity, as in Orzack and Tuljapurkar (1989). However, it is difficult to predict what the interaction may be between this type of selection and selection for reduced demographic stochasticity.

With regard to the impact of population size on the generalizability of the present results, we should note that Shpak (2007) shows that the probability of fixation of a mutation that alters the demographic variance but *λ* holds constant is fully independent of the population size. For example, he validates with simulations the (analytically computed) fixation probability of mutations of this sort by setting *N* = 1000. This generalizes a result by Gillespie (1974), who demonstrates for an unstructured model the independence between the population size and the probability that a mutation with effect limited to the variance in offspring number goes to fixation. Gillespie (1974) explains this apparently paradoxical observation – fixation probabilities typically depend on population size (Crow and Kimura 1970) – by noting that “both the intensity of selection and the magnitude of the stochastic effects are inversely proportional to population size.” Therefore, the results of the present study should hold at any population size, when the environment is constant. However, it should be kept in mind that, in Gillespie's model, the mean time to fixation is proportional to the population size (Gillespie 1974). If this is the case in the age-structured model as well, as Shpak (2007) suggests, then we may expect that in large populations selection against demographic stochasticity may be very slow. The present theory, then, predicts that a higher rate of aging is more likely to be observed in small populations than in large populations, because in small populations, selection for alleles with an opposite effect on survival would proceed more quickly. Interestingly, the same prediction may follow from a different set of considerations. The demographic variance plays a crucial role in the extinction of finite populations. In a constant environment, the ultimate extinction probability of a population is a decreasing function of both the initial population size and the growth rate, but an increasing function of the demographic variance (Engen et al. 2005b). This means that, holding *λ* and the initial population size constant, the probability of extinction is decreased by reducing 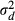. As the present results show that an increase in the rate of aging may imply a reduction in the demographic variance, an increase in the rate of aging may also represent an insurance against extinction. In general, small populations are at higher risk of extinction than big ones. Therefore, this may increase the likelihood of observing a higher rate of aging in small populations than in large populations.

Vindenes et al. (2009) propose that the rate of molecular evolution in a population is a decreasing function of the population demographic variance. Given that the demographic variance depends on the life history of the species, the results of Vindenes et al. corroborate the idea that variation among taxa in the rates of molecular evolution can be explained by variation in life histories (Bromham 2009). Some studies have highlighted that the rate of molecular evolution in mammals is negatively correlated with longevity (Bromham et al. 1996; Nabholz et al. 2008; Welch et al. 2008). The results in the present study seem to be coherent with these findings. If a small demographic variance boosts the rate of molecular evolution (Vindenes et al. 2009), then the negative correlation between the rate of molecular evolution and longevity may be partly explained by the fact that a higher rate of aging may decrease the demographic variance. However, this remains conjecture in the absence of estimates of the amount of demographic stochasticity for these populations. Moreover, the focus of the present article is on differences in survival. The connection between the rate of aging and the demographic variance may be complicated by considerations about reproduction. Species that differ in longevity may also differ in the pattern of fertility. Different reproductive strategies may have a strong impact on the demographic variance. For example, Sæther and Bakke (2000) found that short-lived avian species with large clutches have a higher demographic variance than long-lived species with small clutches. It would, then, be interesting for future work to explore the demographic stochasticity that would be exhibited by life histories with different degrees of aging in both survival and fertility.

To conclude, we should remind ourselves of a classic evolutionary explanation of aging: the pleiotropy theory (Williams 1957). The theory postulates the existence of mutations with a positive effect on early survival but later detrimental consequences. Typically, natural selection places more weight on early-life rather than late-life performances (Hamilton 1966). Therefore, the overall effect of these mutations on fitness is supposed to be positive. Assuming an infinite population, virtually all explicit models of this theory have equated fitness with the growth rate *λ* and studied the effect that pleiotropic mutations have on *λ* (Charlesworth 1980; Rose 1985; Abrams 1993). These models neglect demographic stochasticity. However, in finite populations, selection against demographic stochasticity may be a powerful force (Shpak 2007). In the present study, it is shown that a class of mutations exists of the same sort postulated by the pleiotropy theory that may increase fitness by decreasing the demographic stochasticity. These mutations do so by holding *λ* constant and therefore cannot be accounted for by these earlier models. The identification of such a class of mutations appears, then, to constitute an extension of the pleiotropy theory to finite populations.
